# Genotype–phenotype associations within the Li-Fraumeni spectrum: a report from the German Registry

**DOI:** 10.1186/s13045-022-01332-1

**Published:** 2022-08-16

**Authors:** Judith Penkert, Farina J. Strüwe, Christina M. Dutzmann, Beate B. Doergeloh, Emilie Montellier, Claire Freycon, Myriam Keymling, Heinz-Peter Schlemmer, Birte Sänger, Beatrice Hoffmann, Tanja Gerasimov, Claudia Blattmann, Sebastian Fetscher, Michael Frühwald, Simone Hettmer, Uwe Kordes, Vita Ridola, Sabine Kroiss Benninger, Angela Mastronuzzi, Sarah Schott, Juliane Nees, Aram Prokop, Antje Redlich, Markus G. Seidel, Stefanie Zimmermann, Kristian W. Pajtler, Stefan M. Pfister, Pierre Hainaut, Christian P. Kratz

**Affiliations:** 1 Pediatric Hematology and Oncology, Hannover Medical School, Carl-Neuberg Str. 1, 30625 Hannover, Germany; 2 Department of Human Genetics, Hannover Medical School, Hannover, Germany; 3 Univ. Grenoble Alpes, Inserm 1209, CNRS 5309, Institute for Advanced Biosciences, F38000 Grenoble, France; 4 Department of Pediatrics, Grenoble Alpes University Hospital, Grenoble, France; 5 Division of Radiology, German Cancer Research Center (DKFZ), Heidelberg, Germany; 6 Department of Pediatric Oncology, Hematology and Immunology, Olgahospital, Klinikum Stuttgart, Stuttgart, Germany; 7 Department of Haematology and Oncology, Sana Hospitals, Lübeck, Germany; 8 Paediatric and Adolescent Medicine, University Medical Center Augsburg, Augsburg, Germany; 9 Division of Pediatric Hematology and Oncology, Department of Pediatric and Adolescent Medicine, University Medical Center Freiburg, University of Freiburg, Freiburg, Germany; 10 Department of Pediatric Hematology and Oncology, University Medical Center Hamburg-Eppendorf, Hamburg, Germany; 11 Department of Pediatric Oncology and Hematology, MITERA Children’s Hospital, Athens, Greece; 12 Department of Oncology, University Children’s Hospital Zürich, Zurich, Switzerland; 13 Department of Haematology, Oncology, Cell Therapy, Gene Therapies and Hemopoietic Transplant, IRCCS Bambino Gesù Children’s Hospital, Rome, Italy; 14 Department of Obstetrics and Gynecology, University of Heidelberg, Heidelberg, Germany; 15 Department of Pediatric Hematology/Oncology, Helios Clinic Schwerin, Schwerin, Germany; 16 Medical School Hamburg (MSH), University of Applied Sciences and Medical University, Hamburg, Germany; 17 Department of Pediatric Hematology and Oncology, Children’s Hospital, Cologne, Germany; 18 Pediatric Oncology Department, Otto von Guericke University Children’s Hospital, Magdeburg, Germany; 19 Division of Pediatric Hematology-Oncology, Department of Pediatrics and Adolescent Medicine, Medical University of Graz, Graz, Austria; 20 Pediatric Hematology and Oncology, University Hospital, Frankfurt, Germany; 21 Hopp Children’s Cancer Center Heidelberg (KiTZ), Heidelberg, Germany; 22 Division of Pediatric Neurooncology, German Cancer Research Center (DKFZ) and German Cancer Consortium (DKTK), Heidelberg, Germany; 23 Department of Pediatric Hematology and Oncology, Heidelberg University Hospital, Heidelberg, Germany

**Keywords:** Li-Fraumeni syndrome, *TP53*, Genotype, Phenotype, Cancer predisposition

## Abstract

Li-Fraumeni syndrome (LFS) is a cancer predisposition syndrome caused by pathogenic *TP53* variants. The condition represents one of the most relevant genetic causes of cancer in children and adults due to its frequency and high cancer risk. The term Li-Fraumeni spectrum reflects the evolving phenotypic variability of the condition. Within this spectrum, patients who meet specific LFS criteria are diagnosed with LFS, while patients who do not meet these criteria are diagnosed with attenuated LFS. To explore genotype–phenotype correlations we analyzed 141 individuals from 94 families with pathogenic *TP53* variants registered in the German Cancer Predisposition Syndrome Registry. Twenty-one (22%) families had attenuated LFS and 73 (78%) families met the criteria of LFS. NULL variants occurred in 32 (44%) families with LFS and in two (9.5%) families with attenuated LFS (*P* value < 0.01). Kato partially functional variants were present in 10 out of 53 (19%) families without childhood cancer except adrenocortical carcinoma (ACC) versus 0 out of 41 families with childhood cancer other than ACC alone (*P* value < 0.01). Our study suggests genotype–phenotype correlations encouraging further analyses.

**Supplementary Information:**

The online version contains supplementary material available at 10.1186/s13045-022-01332-1.

## To the editor

Li-Fraumeni syndrome (LFS; OMIM151623) is a cancer predisposition syndrome caused by pathogenic variants (PVs) in the *TP53* tumor suppressor gene and represents one of the best characterized genetic causes of cancer in children and adults [1–4]. The use of modern DNA-sequencing methods has revealed *TP53* germline PVs in individuals who do not meet established clinical LFS criteria, leading to a Li-Fraumeni spectrum classification [5]. We analyzed factors influencing the cancer risk across this spectrum. The overall aim of such studies is to improve risk prediction to inform cancer surveillance.

Founded in 2017, the German Cancer Predisposition Syndrome Registry collects information on genotypes, personal medical details, family histories, and surveillance, as well as a range of biospecimens. The cutoff date for study inclusion for the present analysis was July 31, 2021. Patients with a germline *TP53* PV (pathogenic or likely pathogenic) or with a somatic mosaic *TP53* PV were included. All variants were curated according to *TP53* specific guidelines [6]. Classic LFS criteria [2], Chompret criteria [4] as well as the Li-Fraumeni spectrum classification [5] were assessed. To search for genotype–phenotype correlations we used functional data from Kato [7], Giacomelli [8], Kotler [9] as well as estimated dominant negative effects based on studies by Monti [10] and Dearth [11]. We tabulated the 94 LFS families and applied the Fisher's exact test to analyze whether the phenotypes (1) LFS versus attenuated LFS and (2) occurrence of childhood cancer other than adrenocortical carcinoma (ACC) alone versus cancer free childhood except ACC were associated with specific genotypic/functional *TP53* PV subgroups. A *P* value of < 0.01 was considered statistically significant. Ethics review and informed consent were obtained.

**Fig. 1:**
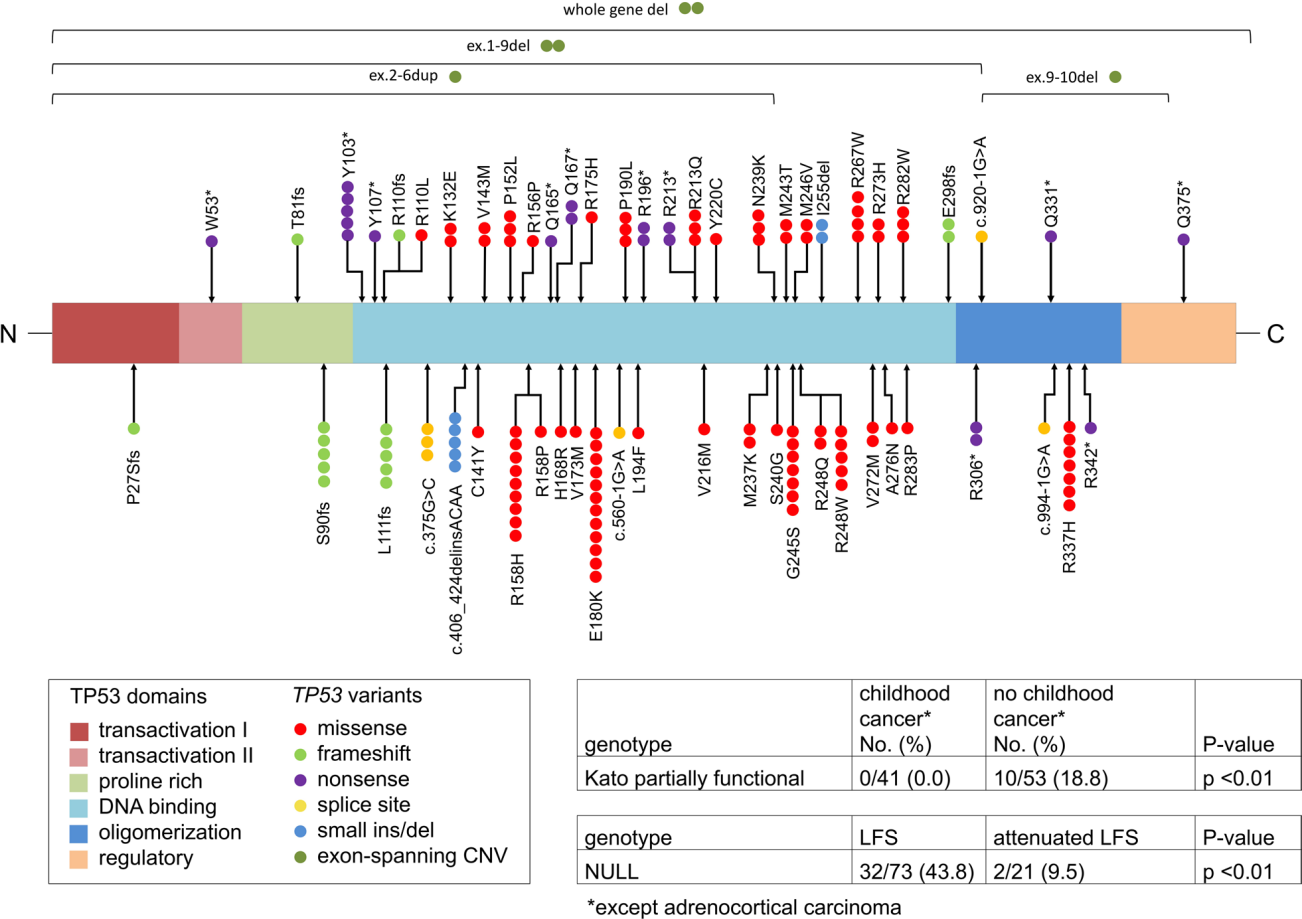
Spectrum of *TP53* germline variants and statistically significant genotype-phenotype correlations. Colored spheres refer to different patients harboring the corresponding variant. *Note*: Y103* is based on two different nucleotide substitutions; whole gene deletions include two gross deletions with differing breakpoints. The genotype–phenotype correlation was based on data from 94 families. CNV, Copy number variation

An overview of all variants, functional data categories, and associated phenotypes including personal and family histories are provided in Additional file 1. The cohort comprises 141 individuals from 94 families; 43 (30.5%) individuals were children or adolescents < 18 years, whereas 98 (69.5%) individuals were adults. There were 98 female and 43 male patients (male-to-female ratio: 0.44). This uneven gender distribution may be due to females being tested more frequently in the context of a breast cancer diagnosis. Four cases with somatic mosaicism were reported. *TP53* PVs as well as statistically significant genotype–phenotype correlations are depicted in Fig. 1.

**Fig. 2:**
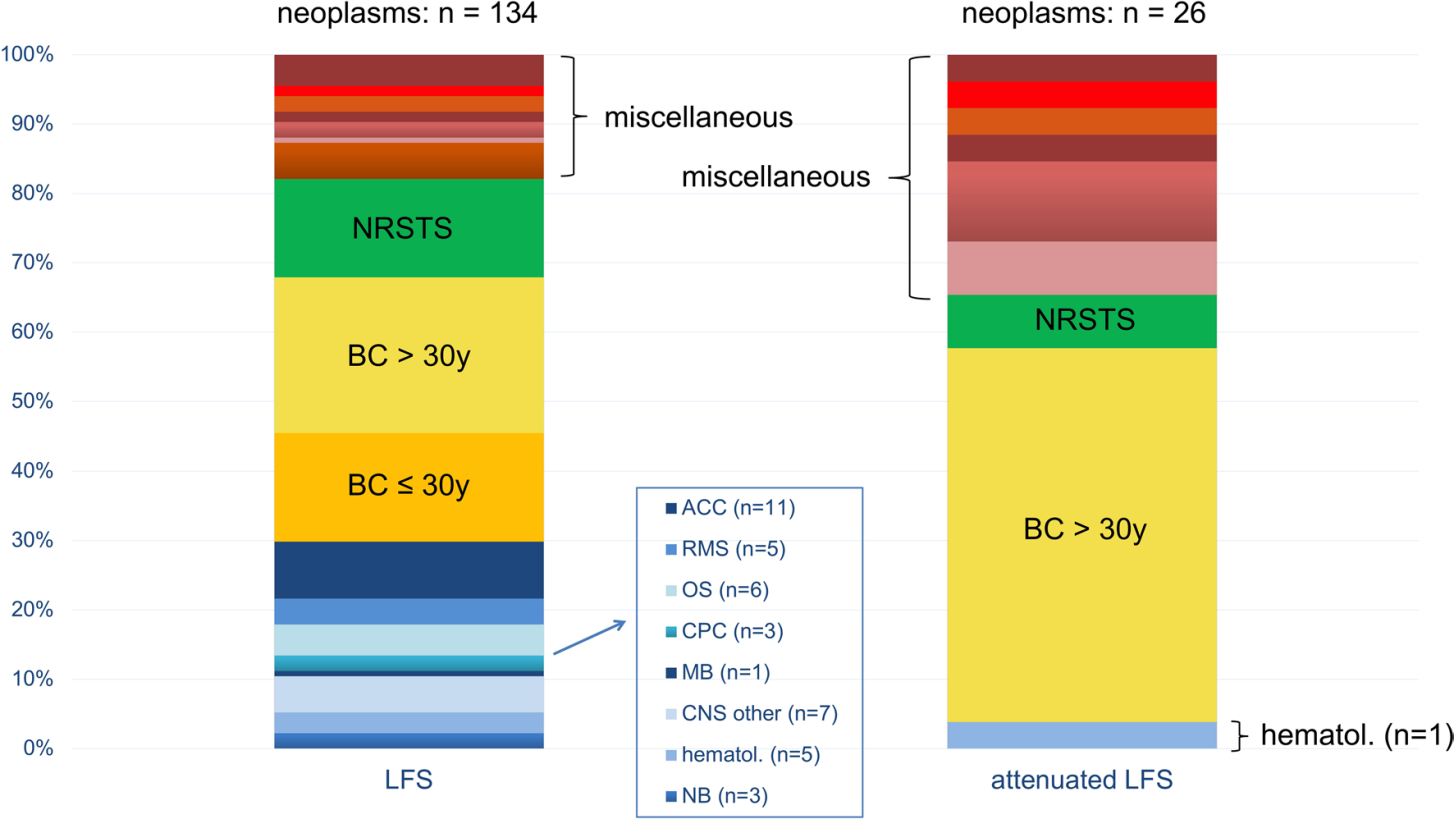
Tumor spectrum in patients with LFS or attenuated LFS. Depicted are all neoplasms reported in the cohort’s individuals (not their families), including subsequent neoplasms occurring in patients with multiple tumors. “Miscellaneous” neoplasms include gastrointestinal, renal, lung, ovarian/tube, melanoma, prostate, and single other (lymphoma, cervical, parotis, basalioma, laryngeal) neoplasms. *ACC* Adrenocortical carcinoma, *BC* Breast cancer, *CML* Chronic myeloid leukemia, *CNS* Central nervous system, *CPC* Choroid plexus carcinoma, *hematol*. Hematological, *MB* Medulloblastoma, *NB* Neuroblastoma, *NRSTS* Non-rhabdomyosarcoma soft tissue sarcoma, *OS* Osteosarcoma, *RMS* Rhabdomyosarcoma

According to the Li-Fraumeni spectrum classification [5], the cohort included 79 individuals with *LFS*, 33 *LFS carriers* as well as 14 individuals with *attenuated LFS* and 15 *attenuated LFS carriers*. No consistent signs of anticipation were observed. In the entire cohort, 33 families (35.1%) did not meet any of the established LFS testing criteria. Thirty-four LFS patients (30.4%) had multiple (between two and five) malignancies, whereas six patients with attenuated LFS (20.7%) had a history of multiple (between two and four) malignancies. Overall, 134 neoplasms occurred in 79 LFS patients, whereas 26 malignancies occurred in 14 individuals with attenuated LFS (Fig. 2). In patients with LFS, breast cancer ≤ 30 years, osteosarcoma, rhabdomyosarcoma, non-rhabdomyosarcoma soft tissue sarcoma, ACC, and central nervous system tumors were diagnosed in 73 of 134 (55%) patients. In individuals with attenuated LFS, more than half of the tumors diagnosed were breast cancers > 30 years. The proportion of miscellaneous neoplasms not known to be strongly associated with *TP53* germline PVs was 34.6% in patients with attenuated LFS compared to 17.9% in patients with LFS. Altogether, 65 breast cancers occurred in the entire cohort, 26 of which were HER2 + , 24 were HER2-, and for 15 tumors histological details were not available.

Kato partially functional variants were statistically significantly associated with a cancer-free childhood, apart from childhood ACC (10 out of 53 families without childhood cancer except ACC versus 0 out of 41 families with childhood cancer except ACC alone, *P* value < 0.01). Typical LFS childhood cancers (i.e., rhabdomyosarcoma, osteosarcoma, choroid plexus carcinoma, medulloblastoma, other brain tumors, and leukemia)—excluding ACC—occurred exclusively in individuals with NULL variants or non-functional missense variants. In general, childhood cancer occurred in more than half of the families with NULL (58.8%) or non-functional missense (52%) variants, whereas in families with partially functional variants ACC was observed as the only childhood cancer, affecting 30% of these families. We observed a statistically significant association between NULL variants and LFS, while this variant type was rare among patients with attenuated LFS: 32 out of 73 families with LFS carried NULL variants, whereas NULL variants were present in two out of 21 families with attenuated LFS (*P* value < 0.01). We did not observe additional statistically significant associations when analyzing the other functional variant subgroups. Case ascertainment, differences in overall survival, family size, and/or family clustering may have introduced a potential bias and represent a limitation of our study.

Despite this limitation, these data suggest that future more detailed genotype–phenotype correlations may allow for accurate cancer risk prediction (time to first malignancy and second cancer risk) and personalized cancer surveillance. Large, international collaboration is required to reach the statistical power to make such risk predictions. Our findings are in agreement with previously published results assessing the correlation between *TP53* genotypes and various other cancer phenotypes in LFS [12, 13]. The observation that a substantial proportion of patients is missed using established LFS testing criteria suggests that the criteria require modification.

## Supplementary Information

**Additional file 1.** TP53 (NM_000546.5) variants, functional data categories, and associated phenotypes. Abbreviations: Acute lymphatic leukemia (ALL), acute myeloid leukemia (AML), adrenocortical carcinoma (ACC), bilateral (bilat), breast cancer (BC), carcinoma (CA), choroid plexus carcinoma (CPC), chronic lymphatic leukemia (CLL), chronic myeloid leukemia (CML), colorectal carcinoma (CRC), ductal carcinoma in situ (DCIS), estrogen receptor (ER), female (f), human epidermal growth factor receptor 2 positive (Her2+), Li-Fraumeni syndrome (LFS), lobular intraepithelial neoplasia (LIN), male (m), medulloblastoma (MB), myelodysplastic syndrome (MDS), neuroblastoma (NBL), non-small-cell lung carcinoma (NSCLC), not available (NA), osteosarcoma (OS), Primitive Neuro-Ectodermal Tumor (PNET), progesterone receptor (PR), rhabdomyosarcoma (RMS), soft tissue sarcoma (STS), triple-negative breast cancer (TNBC). Variants marked * were classified as NULL variants; to reduce complexity, smaller (less than whole exon) deletions were rated as NULL variants as well. The DNE IARC estimation, based largely on studies by Monti and Dearth, was accessed via the TP53 Database (https://tp53.isb-cgc.org).

## Data Availability

All data generated or analyzed during this study are included in this published article and its Additional file 1.
